# High methylation of lysine acetyltransferase 6B is associated with the Cobb angle in patients with congenital scoliosis

**DOI:** 10.1186/s12967-020-02367-z

**Published:** 2020-05-24

**Authors:** Yuantao Wu, Hongqi Zhang, Mingxing Tang, Chaofeng Guo, Ang Deng, Jiong Li, Yunjia Wang, Lige Xiao, Guanteng Yang

**Affiliations:** 1 Department of Spine Surgery and Orthopaedics, Xiangya Hospital, Central South University, Xiangya Road 87, Changsha, 410008 China; 2 National Clinical Research Center for Geriatric Disorders, Xiangya Hospital, Central South University, Xiangya Road 87, Changsha, 410008 China

**Keywords:** Congenital scoliosis, Epigenetic, DNA methylation, Lysine acetyltransferase 6B, RUNX2

## Abstract

**Background:**

The etiology of congenital scoliosis (CS) is complex and uncertain. Abnormal DNA methylation affects the growth and development of spinal development. In this study, we investigated the role of DNA methylation in CS.

**Methods:**

The target region DNA methylation level in the peripheral blood of patients with CS was analyzed. Through in-depth analysis, genes closely related to the growth and development of the vertebra were identified. EdU staining was performed to verify the role of differentially expressed genes in chondrocyte proliferation.

**Results:**

The hypermethylated KAT6B gene was observed in patients with CS, and was positively correlated with the Cobb angle. KAT6B was primarily expressed on chondrocytes. The promoter of KAT6B in CS patients was hypermethylated, and its expression was significantly reduced. Further mechanistic studies revealed that EZH2 mediated trimethylation of lysine 27 on histone H3 of the KAT6B promoter. Overexpression of KAT6B in CS-derived primary chondrocytes can significantly promote chondrocyte proliferation, which may be related to activation of the RUNX2/Wnt/β-catenin signaling pathway.

**Conclusion:**

Epigenetic modification of KAT6B may be a cause of CS. If similar epigenetic modification abnormalities can be detected through maternal liquid biopsy screening, they may provide useful biomarkers for early screening and diagnosis of CS.

## Background

Congenital scoliosis (CS) is caused by abnormal spinal development in the 4th to 6th weeks of pregnancy, resulting in asymmetric spinal growth [1]. CS can be divided into the following three types: Type I: vertebral formation disorders, including hemivertebrae, butterfly vertebrae, wedge-shaped vertebrae; type II: poor vertebral segmentation, including block vertebrae, bone bridge; and type III: mixed type, that is, one side vertebral segmental disorder combined with contralateral vertebral formation disorder [1]. Among these types, vertebral formation disorders and poor segmentation account for approximately 80% of the total, while the mixed type accounts for approximately 20%. Type III spinal deformity usually progresses fastest, followed by type I, and the blocked vertebra (bilateral segmental disorder) does not contain growth plates and progresses most slowly [2].

The incidence of CS in newborns is approximately 0.5% to 1.0%, which is sporadic. The etiology of CS is complex and uncertain [3]. Deficiency in susceptible genes and multiple genes, maternal exposure to carbon monoxide and placental hypoxia, and diabetes can induce or promote the occurrence and progression of vertebral body development disorder [4]. Studies have shown that vertebral body defects are related to chromosomal rearrangements, including trisomy, mosaic and translocation. The LMX1A gene is reported to be a susceptibility gene for vertebral formation disorders [5], and the TBX6 gene may be a susceptible gene for poor vertebral segmentation, rib deformity, and thoracic spinal deformity [6].

In addition, CS is a dynamic process, and its occurrence and progress follow Hueter-Volkmann’s law; in other words, the growth of the epiphysis is suppressed when the pressure is increased, and the growth is accelerated when the pressure of the epiphysis is reduced [7]. With the formation of lateral curvature, the pressure on the concave side of the vertebral epiphyseal plate will be significantly higher than that on the convex side, and the growth speed on the two sides of the concave and convex is imbalanced, which turns the disease progress into a vicious circle [7]. The severity and rate of progression of spinal deformities caused by hemivertebra are clinically difficult to predict, and are related to the type of hemivertebra, the site of occurrence [8]. Early diagnosis and surgical treatment can avoid serious secondary deformities, reduce fusion and fixation segments, and retain more spinal mobility, thereby improving children’s quality of life.

Epigenetics is the result of the interaction between environmental factors and cellular genetic material. Epigenetics is the study of heritable expression changes without DNA sequence changes, including DNA methylation, histone modification, chromatin modification, and RNA interference [8]. At present, the most deeply studied epigenetic mechanism is the methylated form. Changes in the methylation status of DNA are susceptible to reversible modification by external factors such as hormones, diet, and drugs, indicating that the gene is silent or expressed [9]. The higher the degree of methylation is, the more “silent” the gene is, and vice versa; in other words, the level of gene transcription is inversely related to its methylation level. Abnormal DNA methylation patterns can cause a variety of human diseases, including hereditary diseases, tumors, autoimmune diseases, and neuropsychiatric diseases [10, 11]. In the research of congenital diseases, abnormal DNA methylation patterns can cause imprinting dysfunction, which will affect the growth and development of the fetus, leading to the occurrence of genetic diseases, such as Beckwith–Weidemann syndrome (BWS) and Prader-Willi/Angelman syndrome [12–14]. Researchers believe that DNA methylation plays an important role in the differentiation of specific tissues and organs, such as the vertebral column [15].

In this study, from the perspective of epigenetics, the target region DNA methylation level in the peripheral blood of patients with CS was analyzed. Through in-depth analysis of ChIP results, genes closely related to the growth and development of the vertebral body were identified. We also verified the gene expression and the role in chondrocyte proliferation and apoptosis, and explored the possible mechanism of its role in the pathogenesis of CS in combination with epigenetic abnormalities.

## Materials and methods

### Human sample collection

There are two independent sample cohorts in this study. The first cohort included 50 normal healthy individuals and 50 patients with CS collected from September 2016 to September 2018 in Xiangya Hospital. The peripheral blood samples were collected from the first cohort and were used for DNA methylation analysis. The second cohort included 10 controls (non-IS patients with lumbar disk herniation and spine fracture), and 13 patients with CS collected from June 2017 to June 2018. Facet joint tissues were collected from the second cohort and used to test gene expression and isolate chondrocytes. The patients with CS and controls were identified based on their clinical manifestations, X-ray, CT, and MRI. The characteristics of subjects are shown in Table 1. This study was approved by the Ethics Committee of Xiangya Hospital (No. 201703358). Written informed consent was obtained from all patients and controls who participated in the experiments and from their legal guardians following the Helsinki Declaration.

**Table 1:** Characteristics of participants in the studied cohorts

Characteristics	The first cohort		The second cohort	
	Control (N = 50)	CS (N = 50)	Control (N = 10)	CS (N = 13)
Age, years	13.21 ± 6.2	11.16 ± 4.0	12.82 ± 5.73	12.04 ± 3.4
Gender (female/male)	25/25	24/26	5/5	6/7
Cobb angle (^o^)	–	40.58 ± 12.80	–	42.47 ± 10.38

### Target region DNA methylation analysis

Five milliliters of venous blood were collected from the subjects, and EDTA was added for anticoagulation. A SQ Blood DNA Kit II (OMEGA Bio-TEK, USA) was used to extract DNA according to the instructions. The extracted DNA content was quantified with a UV spectrophotometer (BioSpectrometer fluorescence. Eppendoff, Germany). The integrity of the DNA samples was then checked by 1% agarose gel electrophoresis. The qualified DNA was adjusted to 50 mg/L and stored in a − 20 °C refrigerator until use. DNA bisulfite transformation was performed according to the EZ DNA Methylation-Gold Kit (Zymo Research, Tustin, CA, USA). DNA methylation signals were obtained and analyzed using the SureSelectXT Methyl-Seq kit (Agilent, Santa Clara, CA, USA) through Illumina multiplexed sequencing. Two site-specific probes were set up for each methylation site. Two-color fluorescence signals were used to detect methylated and unmethylated alleles. The difference analysis used an empirical Bayes moderated t test in the limma package to test each methylation site probe, and calculated the multiple hypothesis test correction. The sites were selected when they met the following criteria: ①Adjust P < 0.05; ②Beta-Difference of case and control is not less than 0.2. With reference to the differences in methylation of blood DNA samples from the case group and the control group, genes with large differences were selected, and candidate abnormal methylation genes were screened for subsequent verification in conjunction with databases, such as NCBI and PubMed.

### Chondrocyte isolation and culture

The fascia and perichondria were removed from the facet joint tissues. The isolated cartilage tissue was chopped into 0.3–0.5 mm tissue pieces, and transferred into a 25 cm^2^ culture flask, and the cartilage was washed 3 times with a PBS solution. Trypsin (0.25%) was added 10-15 times the volume of cartilage, and digestion was terminated at 37 °C for 2 h. Type II collagenase (0.02%) was used to further digest the tissues at 37 °C overnight. DMEM culture medium was added to dilute type II collagenase to terminate digestion. The cell pellet was later filtered through a 200-mesh filter. The filtrate was collected and centrifuged at 1500 g for 5 min. DMEM with 10% fetal bovine serum was added to resuspend the cells. The cells were cultured at 37 °C with 5% CO_2_ for subsequent experiments.

### Cell transfection

To overexpress KAT6B gene, 1 × 10^6^ primary chondrocytes were seeded in 6-well plates to 80% confluence, and then transfected with lentivirus expressing KAT6B constructed by GenePharma (Shanghai, China) and packaged into the hU6-MCS-PGK-EGFP lentiviral vector (GenePharma, Shanghai, China). The multiplicity of Infection was 100. After 48-h transfection, transfection efficiency was determined by FACSCalibur flow cytometry (Becton–Dickinson, Franklin Lakes, NJ, USA) with a transfection efficiency of over 90%.

### Immunohistochemistry assays

The tissues were cut into 4 μm slides. After blocking with nonfat 5% milk for 60 min at 37 °C, the slides were located with primary antibodies (anti-KAT6B antibody, 1: 200 dilution, cat no. ab191994, Abcam) at 4 °C overnight. The slides were found with appropriate secondary antibodies at 37 °C for 1 h and co-stained with hematoxylin for 2 min at room temperature. The images were acquired using a microscope (Nikon ECLIPSE 80i, Nikon Corporation, Tokyo, Japan).

### RNA isolation and real-time PCR

Total RNA isolation was performed with TRIzol reagent (Invitrogen, USA). Gene expression was measured by real-time PCR according to the manufacturer’s protocol in a CFX96 Real-Time System (Bio-Rad, USA). All primers were synthesized by Sangon Biotech (Shanghai, China) and GAPDH was used as an endogenous control. Primer sequences are shown in Additional file 1: Table S1. The data were analyzed using the comparative Ct (2^−ΔΔCT^) method.

### Western blot analysis

Isolated chondrocytes were collected for protein extraction using RIPA lysis buffer. A BCA Protein Concentration Assay Kit (Thermo Scientific, USA) was used to determine the protein concentration of the sample according to the manufacturer’s protocol. Proteins were separated on a 10% SDS gel and transferred into PVDF membranes. Primary antibodies were used as follows: anti-KAT6B antibody, 1:1000 dilution, cat no. ab191994, Abcam; anti-RUNX2 antibody, 1: 1000 dilution, cat no. ab76956, Abcam; anti-RUNX3 antibody, 1: 1000 dilution, cat no. ab49117, Abcam; anti-COL2A1 antibody, 1: 100 dilution, cat no. ab239007, Abcam; anti-COL10A1 antibody, 1: 1000 dilution, cat no. ab182563, Abcam; anti-β-catenin antibody, 1:1000 dilution, cat no. ab6302, Abcam; anti-c-Myc antibody, 1:1000 dilution, cat no. ab32072, Abcam; anti-VEGF antibody, 1:1000 dilution, cat no. AB-293-NA, Bio-Techne; anti-GAPDH antibody, 1:3000 dilution, cat no. sc-47724, Santa Cruz Biotechnology. After primary antibody incubation, the membranes were incubated with a secondary anti-rabbit antibody or anti-mouse antibody (1: 5000, Cell Signaling Technology, USA). The bands were measured using a chemiluminescence system (Bio-Rad, USA) imaging system.

### Chromatin immunoprecipitation (ChIP)

Primary chondrocytes were isolated from patients with CS and the controls, and cross-linked chromatin was prepared, which was then broken up into fragments of approximately 200 bp in size using a Bioruptor UCD-200 sonicator (Diagenode). These fragmented chromatin samples were incubated with specific antibodies (anti-H3K4me3 antibody, 1:200 dilution, cat no. ab8580, Abcam; anti- H3K9me3 antibody, 1: 200 dilution, cat no. ab8898, Abcam; anti- H3K27me3 antibody, 1: 100 dilution, cat no. ab6002, Abcam; anti- H3K36me3 antibody, 1: 200 dilution, cat no. ab9050, Abcam; anti- H3K79me3 antibody, 1: 200 dilution, cat no. ab2621, Abcam) overnight at 4 °C. Rabbit serum was used as a control. RNA elution and purification were performed using the High-Sensitivity ChIP Kit (ab185913, Abcam) according to the manufacturer’s instructions. The KAT6B promoter was detected by qPCR.

### Dual luciferase reporter gene assay

KAT6B promoter fragments were amplified from human genomic DNA by PCR. Primer sequences are shown in Additional file 1: Table S1. All fragments were inserted into the pGL3-LUC reporter vector (Promega). Corresponding mutants were generated using the Site-directed Mutagenesis Kit (Sangon Biotech (Shanghai) Co., Ltd., Shanghai, China) according to the manufacturer’s instructions. All constructs were verified by DNA sequencing. HEK293T cells (2 × 10^5^) were seeded in a 24-well plate for 12 h, and 100 ng of the blank pcDNA3 vector or the pcDNA3 vector containing the EZH2 cDNA and the 400 ng LUC reporter vector were co-transfected into the cells for 48 h. Co-transfection with the pRL-TK Renilla Luciferase Reporter Vector (Promega) was used as a control. After transfection, cells were harvested and analyzed using Dual Luciferase Assay (Promega) according to the manufacturer’s instructions.

### EdU (5-ethynyl-2′-deoxyuridine) staining

To analyze primary chondrocyte proliferation after transfection, EdU staining was conducted using the BeyoClick EdU Cell Proliferation Kit with Alexa Fluor 594 (Cat. no. C0078S, Beyotime Biotechnology, Shanghai, China,) according to the manufacturer’s instructions. EdU was added to the medium until the final concentration was 10 μM, and the cells were maintained for 2 h. After incubation, the cells were washed with PBS to remove the DMEM and the free EdU probe and then fixed in 4% paraformaldehyde at RT for 15 min. After being co-stained with DAPI, the cells were observed under a Leica fluorescence microscope. EdU positive cells were counted at 5 different areas per sample and reported as a percentage of EdU-positive cells.

## Results

### DNA methylation profiles of CS samples

**Fig. 1:**
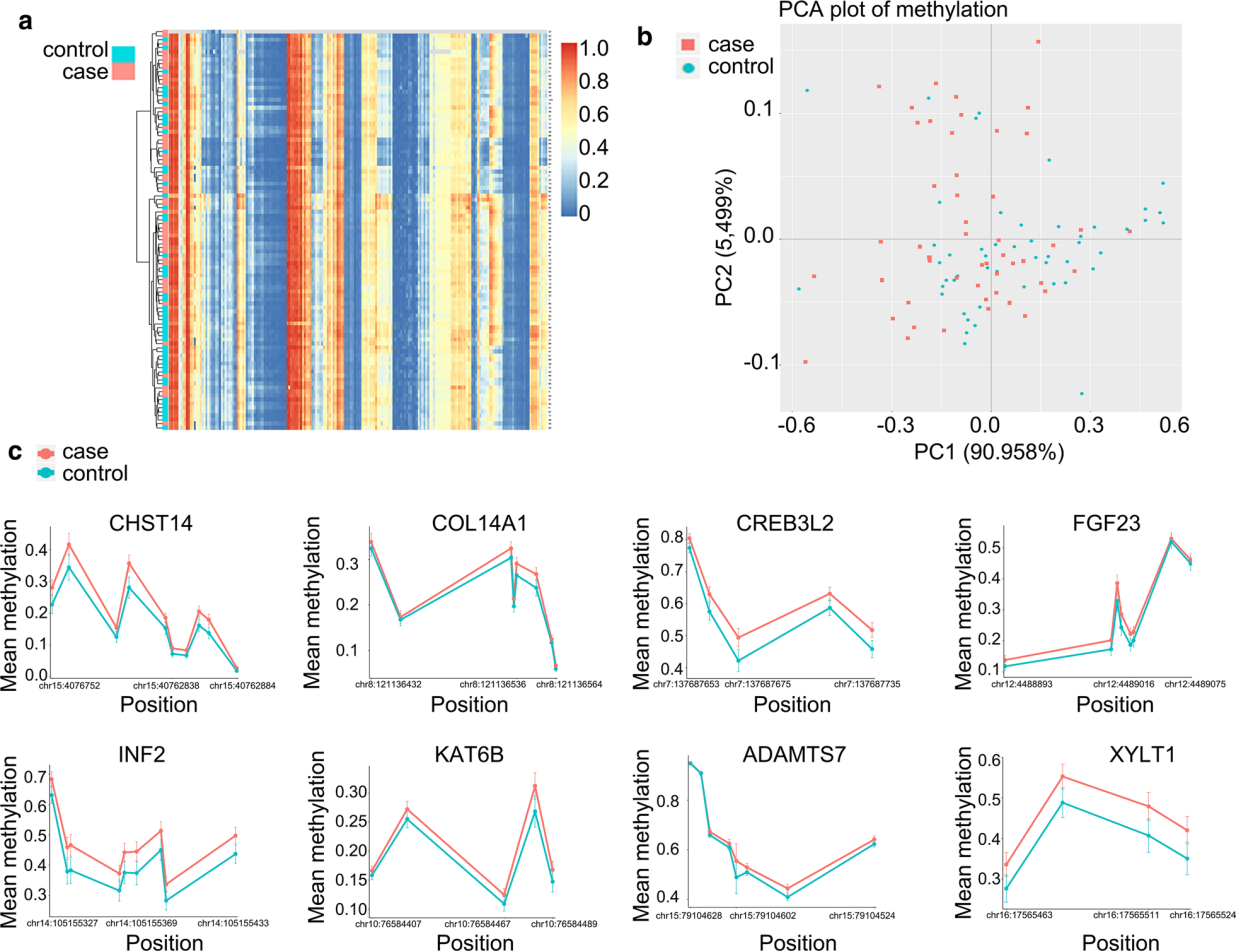
DNA methylation analysis in blood samples from patients with congenital scoliosis. **a** A target region DNA methylation sequencing was performed to screen the differential methylation. Heat map of differential methylation in genes. **b** Principal component analysis (PCA) in congenital scoliosis samples and healthy samples. **c** The mean methylation levels and position of the top eight differentially expressed genes. CHST14, carbohydrate sulfotransferase 14; COL14A1, collagen type XIV alpha 1 chain; CREB3L2, cAMP responsive element binding protein 3 like 2; FGF23, fibroblast growth factor 23; INF2, inverted formin 2; KAT6B, lysine acetyltransferase 6B; ADAMTS7, a disintegrin and metalloproteinase with thrombospondin motifs 7; XYLT1, xylosyltransferase 1

**Fig. 2:**
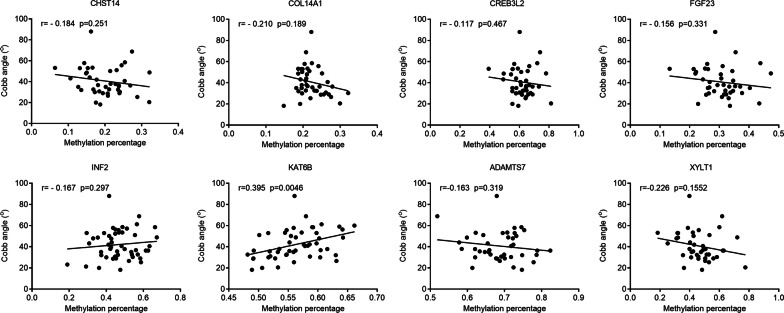
Association of Cobb angles and methylation levels of eight genes, including CHST14, COL14A1, CREB3L2, FGF23, INF2, KAT6B, ADAMTS7 and XYLT1

To study the possible epigenetic pathogenesis of CS, we collected peripheral blood samples from 50 patients with CS and 50 healthy people for methylation analysis. We analyzed the differences in DNA methylation profiles among the 50 CS samples using unsupervised hierarchical clustering based on the results of the target region DNA methylation analysis (Fig. 1a). Then, 212 probes that were aberrantly methylated in the 50 CS samples in comparison with the 50 heathy control samples were identified, indicating that DNA methylation alterations had occurred in CS relative to the normal control (Fig. 1a). When we performed PCA using the DNA methylation levels of these 212 probes, the CS samples showed a DNA methylation profile that differed distinctly from the DNA methylation profiles of heathy control samples (Fig. 2). Eight of the 212 probes were annotated around the transcription start sites (TSSs), such as the region from 200 bp upstream of the TSS to 1500 bp upstream. These eight probes for the CHST14, COL14A1, CREB3L2, FGF23, INF2, KAT6B, ADAMTS7 and XYLT1 genes showed significantly higher methylation levels for at least three CpG sites in CS samples than in healthy controls (Fig. 1c).

We then analyzed the correlation between methylation status and clinical characteristics in CS. As shown in Fig. 2, the methylation level of the KAT6B promoter was positively associated with the Cobb angle in CS patients (Spearman r = 0.395, P = 0.0046). No significant correlation was found between the methylation status of CHST14, COL14A1, CREB3L2, FGF23, INF2, ADAMTS7 and XYLT1 and the Cobb angle (P > 0.05 for all).

### H3K27me3 modification in the KAT6B promoter in the facet joint chondrocytes of CS patients

**Fig. 3:**
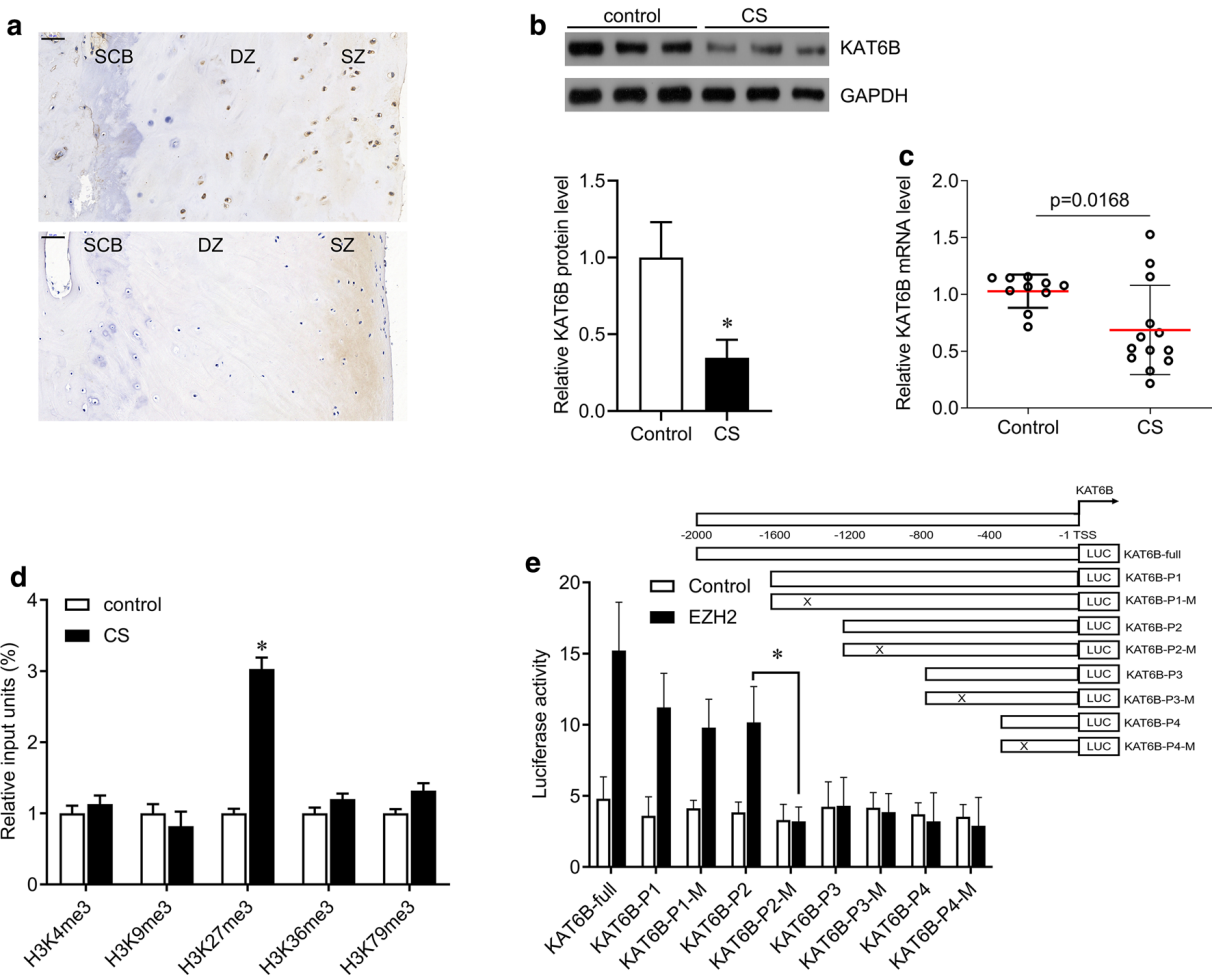
EZH2 mediates trimethylation of H3K27 on KAT6B promoter. **a** Immunohistochemical staining for KAT6B in facet joint tissues. SCB, subchondral bone; DZ, deep zone; SZ, superficial zone. Bar, 50 μm. **b** Western blot was performed to measure the expression of KAT6B in primary chondrocytes (upper), and quantification of the bands (lower). **c** qPCR was performed to measure the expression of KAT6B in primary chondrocytes from control (N = 10) and CS (congenital scoliosis, N = 13) groups. **d** ChIP was performed in primary chondrocytes from control and CS (congenital scoliosis) using the indicated antibodies or IgG, and qRT–PCR was performed to test the promotor of KAT6B. **e** Schematic diagrams of the KAT6B promoter segments and mutations. Numbers indicate the nucleotides relative to KAT6B TSS (transcriptional start site) (upper panels). Luciferase reporter activities of human 293T cells transiently co-transfected with luciferase reporter constructs containing the wild-type sequence of KAT6B promoter or its mutant counterparts, together with EZH2 for 48 h

We next further analyzed the expression of KAT6B in the facet joint tissues of CS patients. By IHC staining, we found that KAT6B primarily expressed in chondrocytes of the surface zone, and KAT6B-positive cells were significantly reduced in CS samples compared with the control group (Fig. 3a), suggesting that KAT6B plays a role in chondrocytes. We then isolated chondrocytes from facet joint tissues to measure the protein and mRNA levels of KAT6B. The expression of KAT6B in chondrocytes of CS patients was significantly downregulated at the protein and mRNA levels compared with control group (Fig. 2b, C). High methylation was found in the KAT6B promoter (Fig. 1c). We then tested the mechanism underlying the high methylation of KAT6B byChIP. We found that H3K27me3 but not H3K4me3, H3K9me3, H3K36me3 and H3K79me3, was highly expressed in KAT6B promoter in chondrocytes of CS patients (Fig. 3d). The histone methyltransferase enhancer of zeste 2 (EZH2) is the enzymatic catalytic subunit of polycomb repressive complex 2 (PRC2) and is involved in transcriptional repression by specifically trimethylating lysine 27 on histone 3 (H3K27) [16]. Therefore, we performed a luciferase assay to test the binding site of EZH2 in KAT6B promoter. The promoter sequences (including full length, truncate type and mutant type) were constructed, and a luciferase assay was performed. We found that the luciferase activity was significantly increased in KAT6B-full (2000 bp upstream of TSS), KAT6B-P1 (1600 bp upstream of TSS), KAT6B -P1-M, KAT6B-P2 (1200 bp upstream of TSS), but reduced in KAT6B-P2-M, KAT6B-P3 (800 bp upstream of TSS), KAT6B-P3-M, KAT6B-P4 (400 bp upstream of TSS), KAT6B-P4-M (Fig. 3e), indicating that EZH2 bound to the KAT6B promoter 1200–800 bp upstream of TSS.

### Expression of RUNX signaling in the facet joint chondrocytes of CS patients

**Fig. 4:**
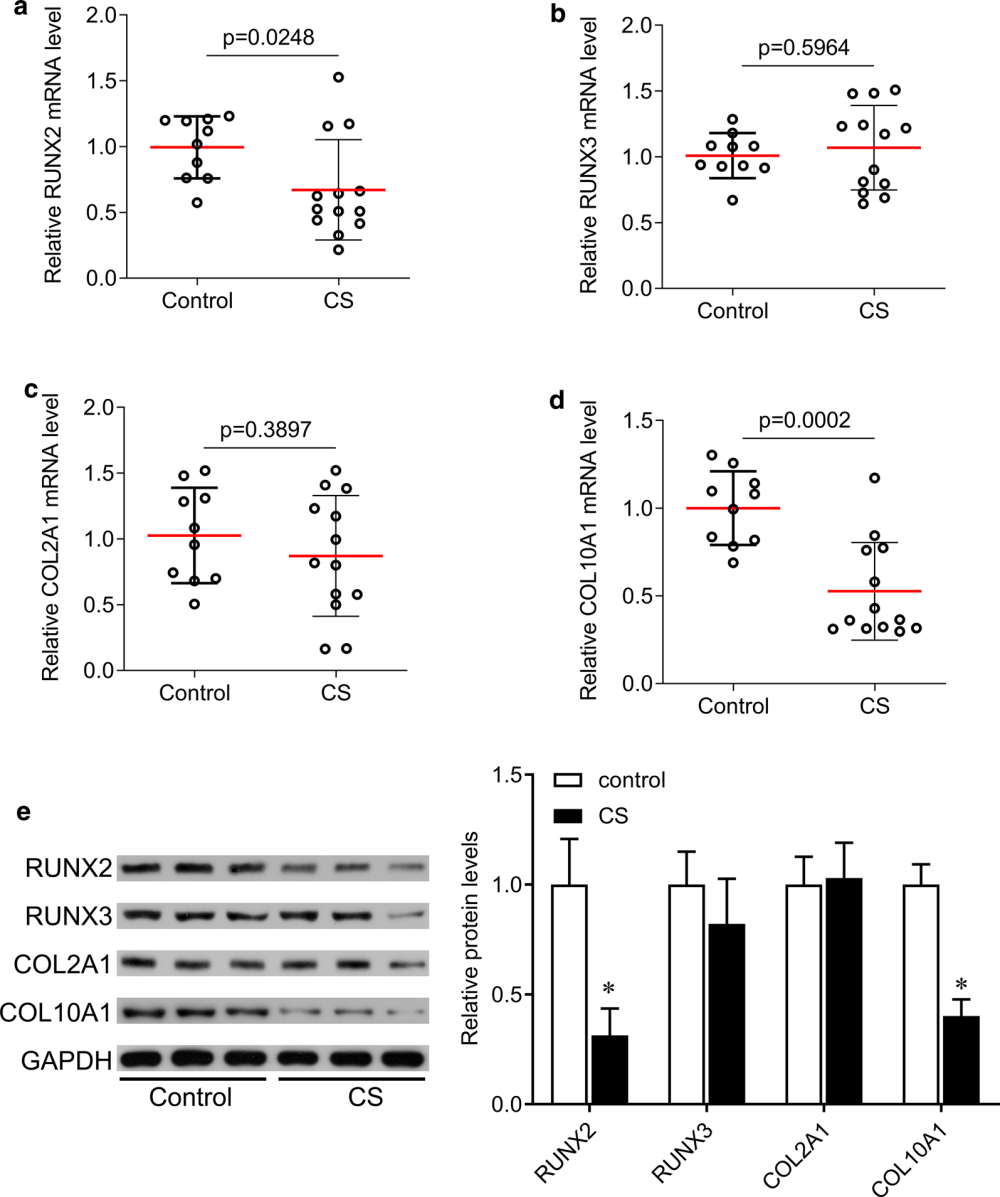
Expression of KAT6B downstream molecules. qPCR was performed to measure the expression of RUNX2 (**a**), RUNX3 (**b**), COL2A1 (**c**), and COL10A1 (**d**) in primary chondrocytes from control (N = 10) and CS (N = 13) groups. **e** Western blotting was performed to measure the expression of RUNX2, RUNX3, COL2A1, and COL10A1 in primary chondrocytes from control and CS groups. CS, congenital scoliosis. *p < 0.05

KAT6B is known as an inducer of RUNX signaling, which is related to chondrocyte proliferation. We tested the expression of RUNX2, RUNX3, COL2A1 and COL10A1 in primary chondrocytes from CS patients. The results showed that the expression of RUNX2 and COL10A1 was significantly downregulated in CS patients compared with control group. There were no significant alterations in RUNX3 and COL2A1 levels (Fig. 4).

### Overexpressing KAT6B promotes cell proliferation in primary chondrocytes from CS patients

**Fig. 5:**
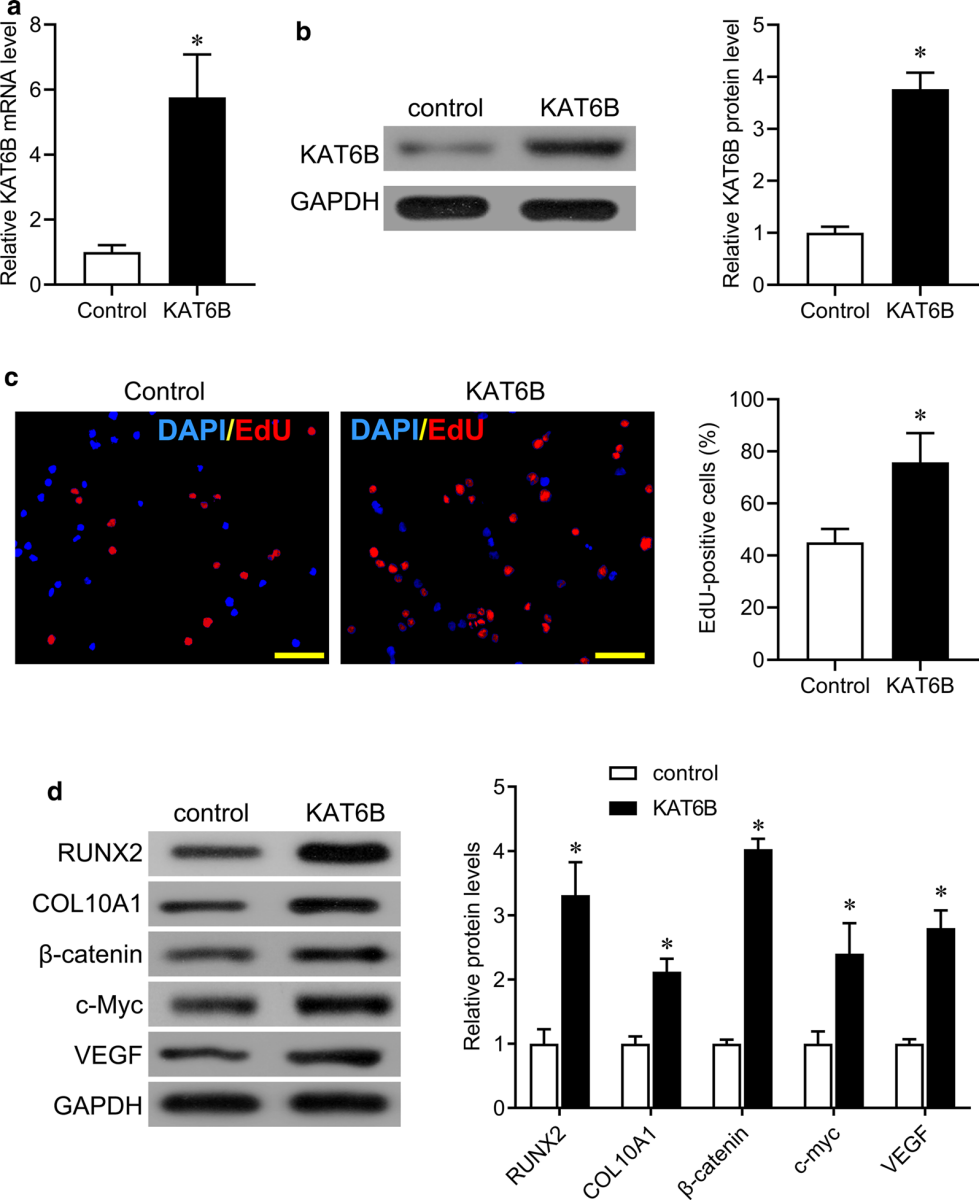
KAT6B promotes chondrocyte proliferation. **a** qPCR was performed to measure the expression of KAT6B in primary chondrocytes from CS tissues after transfection. **b** Western blot was performed to measure the expression of KAT6B in primary chondrocytes from CS tissues after transfection. **c** EdU staining was performed to test cell proliferation in primary chondrocytes from CS tissues after transfection. DAPI, blue; EdU, red. Bar, 100 μm. **d** Western blotting was performed to measure the expression of RUNX2, COL10A1, β-catenin, c-Myc, and VEGF in primary chondrocytes in primary chondrocytes from CS tissues after transfection. CS, congenital scoliosis. *p < 0.05

To investigate the effect of elevated levels of KAT6B on chondrocytes, we overexpressed KAT6B in primary chondrocytes obtained from CS patients. Successful overexpression of KAT6B was observed at the mRNA and protein levels (Fig. 5a, b). Additionally, EdU staining results showed that EdU-positive cells were significantly increased in KAT6B-overexpressing primary chondrocytes compared with control cells (Fig. 5c). Furthermore, the levels of KAT6B downstream molecules, including RUNX2, COL10A1, β-catenin, c-Myc and VEGF, were significantly increased in KAT6B-overexpressing primary chondrocytes (Fig. 5d). Taken together, these results suggest that EZH2-mediated H3K27m3 on the KAT6B promoter decreases the expression of KAT6B, which could further decrease the expression of RUNX2 and inactivate Wnt/β-catenin signaling, ultimately impacting chondrocyte proliferation and spinal growth.

## Discussion

The etiology of CS has not been fully elucidated. The interaction between multigene heredity and environmental factors is the main cause of spinal developmental defects [17]. Understanding the genetic factors and epigenetic regulatory mechanisms of CS may facilitate early prenatal screening for CS, and provides a reliable basis for early diagnosis and treatment. In this study, methylation analysis of peripheral blood genomes of patients with CS revealed that the KAT6B gene was hypermethylated, and the methylation level was positively correlated with the Cobb angle. DNA methylation modification, as an important part of epigenetic regulation, has a significant effect on the normal development of embryos, and its abnormal modification causes vertebral dysplasia [18]. People’s daily exposure to some environmental factors that are harmful to health, and nutritional factors can increase genetic instability and change the cell material metabolism. Changing the dynamic balance of DNA methylation patterns has serious consequences on embryo and fetal development [19]. Epigenetics reveals how environmental factors affect the selective expression of genes. Molecular studies of epigenetic regulation of CS, which is difficult to explain by traditional genetics, will provide new scientific evidence for the active and effective prevention of vertebral defects in fetuses. Therefore, our future work may explore whether analysis of maternal peripheral blood genomic methylation can predict the occurrence of CS and enable early diagnosis and treatment.

KAT6B (lysine acetyltransferase 6B) gene is located in the 10q22.2 region, which contains 18 exons and encodes 2073 amino acids. The encoded protein is part of a histone acetyltransferase and MOZ/MORF protein complex [20]. The N-terminus of KAT6B is involved in transcriptional activation, while its C-terminus is involved in transcriptional repression activity. KAT6B is necessary for RUNX2-dependent transcriptional activation, and is essential for the early developmental regulation of the bone and nervous system [21]. The heterozygous mutation of the KAT6B gene can cause SBBYSS (Say-Barber-Biesecker-Young-Simpson syndrome), a rare form of mental retardation and related syndromes, which is mainly manifested as severe mental retardation, special facial features, bone and genital abnormalities [22]. The KAT6B gene mutation also causes Genitopatellar syndrome, which is mainly characterized by flexion contractures of the hip and knee joints, and ankle foot deformities [23]. In addition, the location of the KAT6B mutation is related to the patient’s phenotype. In patients with SBBYSS syndrome, mutations occur more distally to exon 18 and lack the transcriptional activation domain that causes loss of function mutations. Studies have also shown that exon variation is closer to the typical characteristics of SBBYSS [24]. Therefore, abnormal expression of KAT6B gene and epigenetic modification disorders are closely related to bone development. This study found that KAT6B was mainly expressed on chondrocytes, and the promoter of KAT6B in CS patients was hypermethylated and its expression was significantly reduced. Trimethylation of H3K27 induces transcriptional repression, and thereby participates in controlling gene expression patterns. EZH2 is a methyltransferase and component of polycomb repressive complex 2 (PRC2) and plays an essential role in the epigenetic maintenance of the H3K27me3 repressive chromatin mark. Abnormal EZH2 expression has been associated with the regulation of bone development [25, 26]. In this study, we here revealed that EZH2 mediated H3K27 trimethylation of KAT6B promoter. Overexpression of KAT6B in CS-derived primary chondrocytes can significantly promote chondrocyte proliferation, which may be related to activation of the RUNX2/Wnt/β-catenin signaling pathway. Runx2 is a major determinant of osteoblast differentiation and regulates chondrocyte proliferation, differentiation, and hypertrophy during bone formation in cartilage [27]. The Wnt/β-catenin signaling pathway is an important signaling pathway for bone metabolism, that regulates bone growth [28]. Classical Wnt/β-catenin is closely related to chondrocyte hypertrophy. Many studies have revealed the core role of the Wnt signaling pathway in maintaining osteoblastic homeostasis, promoting high Runx2 expression during chondrocyte hypertrophy, and activating downstream target genes, including c-Myc and VEGF [29, 30].

## Conclusion

This study found that KAT6B is hypermethylated in CS patients through target region methylation analysis, which is positively correlated with the severity of CS, and determined that EZH2 mediates trimethylation of H3K27 on KAT6B promoter, which is involved in vertebral development via the RUNX2/Wnt/β-catenin signaling pathway. Epigenetic modification of KAT6B may be a cause of CS. If similar epigenetic modification abnormalities can be detected through maternal liquid biopsy screening, they may provide useful biomarkers for the early screening and diagnosis of CS.

## Supplementary information

**Additional file 1: Table S1.** Primer sequences used in this study.

## Data Availability

The datasets used and/or analysed during the current study are available from the corresponding author on reasonable request.
