# Supplementary material for: High methylation of lysine acetyltransferase 6B is associated with the Cobb angle in patients with congenital scoliosis
Source: J Transl Med. 2020 May 24;18:210. doi: 10.1186/s12967-020-02367-z (PMC7245753; doi:10.1186/s12967-020-02367-z)
Supplement: Supplementary file 1 — Additional file 1: Table S1. Primer sequences used in this study. [file 12967_2020_2367_MOESM1_ESM.docx]

Additional file 1: Table S1 Primer sequences used in this study

| KAT6B promoter fragments | forward (5'->3') | reverse (5'->3') |
| --- | --- | --- |
| KAT6B -full | GGGACAGACTTCAGGCAACA | GGCGAAGTTCGGTTTTCCAG |
| KAT6B -P1 | CCTGGGAGGGAAACTTCGTG | TGGCGAAGTTCGGTTTTCCA |
| KAT6B -P2 | ATGAGGCTGCAGGAGTCAAC | TGAGCACGAAGTTTCCCTCC |
| KAT6B -P3 | GGGACAGACTTCAGGCAACA | GCGAAGTTCGGTTTTCCAGG |
| KAT6B -P4 | CCTTGCCAGCATCCCATTCA | TTAGCCAGCGCTGTAAGGAA |
| KAT6B | CGGGTGGCCTTAAGTGTCTT | CCGCATGGCAGATTCTCTCT |
| RUNX2 | TGCTTCATTCGCCTCACAAA | CCCGCCATGACAGTAACCAC |
| RUNX3 | ATTCATTCATTCCCCGTGGC | AAGCGAAGGTCGTTGAACCT |
| COL2A1 | GTAGAGACCCGGACCCGC | ACTCTCCGAAGGGGATCTCA |
| COL10A1 | AACTCCCAGCACGCAGAATCC | TCCCTACAGCTGATGGTCCC |
